# Fault Diagnosis Method for Boost Chopper of High-Speed Maglev Train Based on Deep Time-Series Modeling

**DOI:** 10.3390/s26144393

**Published:** 2026-07-10

**Authors:** Shuhuai Wang, Xin Zhang, Wenxin Wang, Yi Tian, Xindong Wang

**Affiliations:** 1 School of Mechanical, Electronic and Control Engineering, Beijing Jiaotong University, Beijing 100044, China; 24126106@bjtu.edu.cn (S.W.); 25126353@bjtu.edu.cn (W.W.); 2 State Key Laboratory of High-speed Maglev Transportation Technology, CRRC Qingdao Sifang Co., Ltd., Qingdao 266111, China

**Keywords:** high-speed maglev train, boost chopper, fault diagnosis, TimesNet, Convolutional Neural Network

## Abstract

The boost chopper (HS) is a core electrical component of the 440 V grid in high-speed maglev trains, providing reliable power for battery charging and auxiliary systems. Fault diagnosis of the HS is crucial for identifying operational faults and ensuring stable train operation. However, HS faults exhibit both long-period fluctuations and transient characteristics, which are difficult for a single network to capture synchronously. This paper proposes a multi-scale fault diagnosis method based on a TimesNet-CNN dual-branch architecture, constructing a parallel and complementary feature extraction mechanism. The TimesNet branch uses Fast Fourier Transform (FFT) to adaptively identify dominant periods, reshaping the 1D sequence into a 2D structure to explicitly model the global evolution of intra-period fluctuations and inter-period trends via Inception convolution. Meanwhile, the CNN branch employs stacked small convolutional kernels and hierarchical downsampling to extract local high-frequency anomaly features. After feature fusion, the method achieves synergistic discrimination of global periodicity and local transiency. Finally, experiments were conducted on a real-world dataset containing 11 system states (10 fault types and 1 normal state). Experimental results show that the proposed method outperforms TimesNet, CNN, ResNet and Informer models in precision, recall and F1-score. This validates the effectiveness of the dual-branch feature fusion mechanism in capturing multi-scale fault features, achieving high-precision identification of HS faults.

## 1. Introduction

The boost chopper (HS) is a core electrical component of the 440 V power grid in high-speed maglev trains. Its primary function is to stabilize, boost and maintain the DC bus voltage at 440 V through high-frequency chopping control, utilizing AC power from the linear induction generator (LIG) and DC power from the external power supply rails. This provides reliable electrical energy for battery charging and the train’s auxiliary systems [1,2,3,4]. As a key power conversion device within this system, the boost chopper (HS) directly determines the dynamic response characteristics and power supply quality of the onboard grid system [5]. Therefore, conducting research on fault diagnosis methods for the boost chopper not only helps to enhance the reliability and maintainability of the onboard power grid system but also provides technical support for the fault diagnosis and intelligent operation and maintenance (O&M) of key components in high-speed maglev trains.

Fault diagnosis methods can generally be categorized into three types: signal processing-based, model-based and data-driven approaches [6]. Signal processing-based methods achieve diagnosis by analyzing the characteristic changes caused by faults in the time–frequency domain. While they do not require a system model and are easy to implement, strategies such as wavelet–fuzzy fusion [7] and the combination of Laplace wavelets with genetic algorithms and neural networks [8] have proven effective in extracting features from non-stationary signals. However, these methods lack mechanistic support and have limited interpretability [9]. Model-based methods utilize mathematical system models to generate residuals (such as reconstructing inputs based on differential flatness [10] or predicting the levitation gap using Kalman filtering [11]). These methods offer high sensitivity and strong interpretability but rely heavily on precise modeling, making them difficult to apply to structurally complex systems. In contrast, data-driven methods extract fault patterns directly from historical data without requiring explicit system models, demonstrating significant advantages in complex dynamic scenarios.

In recent years, deep learning-based methods have been increasingly applied to time-series fault diagnosis. Attention-enhanced CNN models have been used for motor fault diagnosis under small-sample conditions, while optimized LSTM networks have been applied to converter transformer fault diagnosis, owing to their capabilities in local feature extraction and temporal dependency modeling, respectively [12,13]. More recently, attention mechanisms, Transformer models, and hybrid deep learning architectures have further improved the representation capability of diagnostic models. Simulation data-driven fault diagnosis methods based on attention mechanisms and transfer learning have been proposed for rolling bearing monitoring under cross-condition and cross-location scenarios. By combining simulated fault signals, self-attention-assisted feature extraction and distribution alignment, such methods can alleviate the lack of real fault data and improve cross-domain diagnostic robustness [14]. Improved attention-mechanism-based Transformer models have also been applied to time-series anomaly detection, where enhanced attention mechanisms are used to capture correlations among different time points and long-distance temporal dependencies [15]. In addition, hybrid architectures such as DWT-1D-CNN-LSTM have been developed for rotating-machinery fault diagnosis, in which DWT is used to enhance time–frequency features, 1D-CNN extracts local features and LSTM captures temporal dependencies [16]. These studies indicate that attention-based, Transformer-based and hybrid deep learning methods have strong potential for complex time-series diagnosis tasks. However, the above methods mainly focus on motor, transformer, and rotating machinery fault diagnosis, general anomaly detection, or cross-domain transfer diagnosis, and many of them rely on single-path temporal modeling, serial feature extraction, or direct feature stacking. They do not explicitly decouple the long-period evolution and short-term transient disturbance characteristics of HS electrical signals.

Currently, fault diagnosis for maglev trains is predominantly focused on the levitation system. ZHANG et al. proposed a fault diagnosis method combining a Self-Organizing Map (SOM) and a BP neural network. By utilizing the SOM for feature clustering and the BP network for precise classification, this approach effectively overcomes the limitations of single networks, demonstrating superior stability and accuracy in the fault diagnosis of levitation controllers for medium-speed maglev trains [17]. WANG et al. introduced a fault diagnosis method for the maglev train levitation system that fuses model-driven and data-driven approaches. Through the synergistic optimization of residual generation and data learning, along with the introduction of a secondary confirmation mechanism for fault isolation results and a fault-data post-processing strategy, this method effectively enhances diagnostic reliability and analytical capability [18]. WU et al. proposed a fault diagnosis model integrating a Multi-Scale Transformer network. By employing multiple convolutional kernels to capture the temporal features of suspension system data, this model successfully achieves fault diagnosis for suspension control [19].

In summary, current research on fault diagnosis for maglev trains exhibits notable limitations. First, HS faults often manifest as complex nonlinear dynamic behaviors, making it difficult for traditional methods based on single-variable thresholds or static features to effectively model their temporal evolution patterns. Second, the research focus is heavily concentrated on the levitation control system and a systematic investigation into the fault diagnosis of the HS has yet to be reported. To address these issues, this paper proposes a TimesNet-CNN deep architecture based on accessible electrical quantities from real-vehicle operational data (including battery current, battery voltage, HS output current, LIG average current, external power supply module DC current and bus voltage). On one hand, the TimesNet branch detects dominant periods via Fast Fourier Transform (FFT) and models them through convolutions in a 2D view to capture long-range periodicity. On the other hand, a lightweight CNN branch extracts local transient features. By concatenating and fusing the periodic and local features, followed by MLP nonlinear mapping and dropout regularization, the proposed method achieves precise end-to-end multi-class fault classification.

The main contributions of this study are summarized as follows:

First, this study introduces a fault diagnosis framework specifically designed for the boost chopper of high-speed maglev trains. Different from existing studies that mainly focus on the levitation system or general power electronic fault diagnosis tasks, this work establishes an HS-oriented diagnostic framework based on real-vehicle operational electrical data, providing a dedicated solution for multi-state diagnosis of the onboard 440 V grid.

Second, this study proposes a complementary multi-scale temporal modeling strategy rather than a simple engineering combination of existing neural network modules. The TimesNet branch is used to model long-period evolution by identifying dominant periodic components and constructing two-dimensional periodic representations, while the CNN branch directly extracts short-term transient disturbances from the raw time-series signals. In this way, the proposed framework explicitly decouples global periodic characteristics and local transient characteristics, which distinguishes it from general hybrid deep learning methods that mainly rely on serial feature extraction or direct feature stacking.

Third, a feature-level fusion and classification mechanism is designed to align and integrate the global periodic features and local transient features in a unified representation space. The introduction of temporal attention further enables the model to emphasize time periods with stronger diagnostic relevance. Ablation experiments and comparative experiments on 11 system states are conducted to verify the contribution of each branch and the superiority of the proposed framework over representative baseline models.

The remainder of this paper is organized as follows. Section 2 introduces the structure, working principle and typical system states of the boost chopper in high-speed maglev trains. Section 3 presents the proposed TimesNet-CNN dual-branch fault diagnosis method, including the TimesNet branch, CNN branch, feature fusion mechanism and classification decision process. Section 4 describes the experimental dataset, sample construction strategy and model training settings. Section 5 analyzes the experimental results of the proposed dual-branch model and further verifies its effectiveness through ablation experiments and comparative experiments. Finally, Section 6 summarizes the main conclusions of this study.

## 2. Fault Analysis of the Boost Chopper in High-Speed Maglev Trains

### 2.1. Structure and Principle of the Boost Chopper in High-Speed Maglev Trains

**Figure 1:**
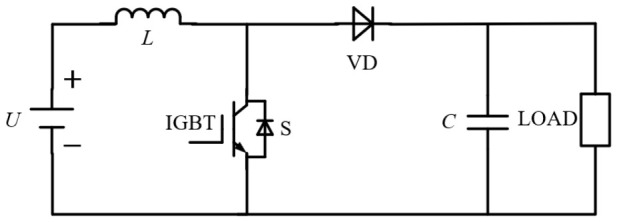
Boost circuit diagram. U denotes the input DC voltage, L denotes the energy-storage inductor, S denotes the IGBT switching device, VD denotes the boost diode, C denotes the output filter capacitor, and LOAD denotes the load.

The boost chopper (HS) of the high-speed maglev train adopts a five-phase parallel boost topology. It is mainly composed of IGBT switches, freewheeling diodes, filter capacitors and energy-storage inductors and is jointly powered by five linear induction generators (LIGs) and one external power supply module. Specifically, the input power for the first four phases comes from the onboard LIGs, while the fifth phase receives input from both the LIG and the external power supply module. Since the HS is essentially a DC-DC converter, the AC power output from the LIGs must first be converted into DC through a rectification stage before undergoing the subsequent boosting process. The output terminals of the five-phase boost circuits are connected in parallel to the same DC bus, where the output currents of each phase converge to collectively supply power to the loads. The circuit diagram of the boost converter is shown in Figure 1.

The working principle of the HS is based on high-frequency Pulse-Width Modulation (PWM) control, with a typical switching frequency of approximately 10 kHz. The IGBTs in each phase are periodically turned on and off under the drive of PWM signals, working in conjunction with inductor energy storage and capacitor filtering to achieve the boost conversion of the input voltage [20]. The system dynamically switches power supply modes according to the train’s operating speed: when the train speed is below 20 km/h, the HS is powered exclusively by the external power supply module; when the speed is in the range of 20–100 km/h, both the linear induction generators (LIGs) and the external power supply module supply power collaboratively; and when the speed exceeds 100 km/h, the system relies entirely on the LIGs to provide electrical energy [21].

### 2.2. System States of the HS

To address the operational safety requirements of the boost chopper (HS) in high-speed maglev trains under complex working conditions, this paper constructs a multi-class diagnostic system comprising 11 system states, including 1 normal operating state and 10 typical fault states. Specifically, F1 represents the normal operating state, while F2–F11 correspond to different abnormal or fault states of the HS and related electrical components. The types of system states are presented in Table 1.

## 3. Materials and Methods

### 3.1. Overall Model Architecture

The TimesNet-CNN dual-branch classification model is a hybrid deep learning framework tailored for multivariate time-series fault diagnosis. Its core idea is to jointly model the global periodic evolution and local transient dynamics of HS fault signals. Traditional single-architecture models, such as pure CNN or pure TimesNet, often struggle to simultaneously capture long-range periodic dependencies and short-term abnormal disturbances. To address this problem, the proposed model adopts a parallel dual-branch structure. For each multivariate time-series sample, the TimesNet branch identifies dominant periodic components and extracts global periodic representations, while the CNN branch directly extracts local transient features from the raw input sequence through one-dimensional convolutional operations. After feature extraction, the global periodic feature and the local transient feature are concatenated along the feature dimension and further fused by a lightweight MLP module. Finally, the fused feature is fed into the classification head to obtain the diagnostic probability distribution of different system states.

Although the basic modules used in this study, such as TimesNet, CNN, temporal attention and MLP, are derived from existing deep learning theories, the proposed method makes task-oriented improvements for boost chopper fault diagnosis. First, the parallel dual-branch architecture is constructed to separately model the two typical characteristics of HS fault signals: long-period evolution and short-term transient disturbance. Second, a temporal attention mechanism is introduced after the TimesNet branch to adaptively aggregate temporal features and highlight key periods with stronger discriminative information, rather than treating all time steps equally. Third, the global periodic features and local transient features are aligned in a unified feature space and fused through a lightweight MLP module, enabling complementary feature-level fusion for final fault classification. Therefore, the improvement of the proposed method lies not in modifying a single basic network module, but in constructing a complementary multi-scale diagnostic architecture tailored to the dynamic fault characteristics of the boost chopper.

### 3.2. TimesNet Model

TimesNet is a deep learning framework specifically designed for time-series modeling, capable of explicit modeling and efficient feature extraction of multi-scale periodic structures within time series. The entire model is trained in an end-to-end manner, enabling it to simultaneously learn period identification, two-dimensional structure construction and feature extraction, thereby demonstrating high adaptability and expressive power [22,23].

#### 3.2.1. Temporal Variations

Before being fed into the proposed network, the continuous operational signals are reconstructed into fixed-length multivariate time-series samples by a sliding-window strategy. Each processed sample consists of *T* consecutive time steps and *C* electrical parameter channels and is used as the input sequence of the model. The detailed sample construction process is described in Section 4.1. Let the processed input sample be denoted as ***X***_1D_. In this study, ***X***_1D_ is organized as a two-dimensional time-channel representation, where each row corresponds to a time point and each column corresponds to a measured variable.

Within the TimesNet branch, period detection is performed during network forward propagation. For the input representation ***X***_1D_, the FFT-based period detection operation is first applied independently to each variable channel to obtain the complex frequency spectrum, and the corresponding amplitude is then calculated. To enhance the robustness of period detection, the amplitudes of all variable channels are averaged along the channel dimension:

(1) $$A=\mathrm{Avg}(\mathrm{Amp}(\mathrm{FFT}(X_{1D})))\in {\mathbb{R}}^{T}$$

where Amp is the amplitude function and Avg is the arithmetic mean along the channel dimension. Through this averaging operation, noise interference from individual channels is suppressed, thereby highlighting the periodic patterns common to the entire system.

After obtaining the spectral amplitude *A*, the top *k* frequency components with the highest energy are extracted to identify the dominant frequencies. The corresponding period lengths are then calculated according to the relationship between frequency and period. The specific steps are as follows:

Given that the FFT result of a real-valued time series exhibits conjugate symmetry, the latter half of the frequency spectrum contains redundant information and may introduce high-frequency noise. Therefore, only the first half of the frequency spectrum is retained for analysis.

Frequency selection: From the retained frequency components of *A*, the indices of the top *k* components with the highest energy are selected:

(2) $$\left\{f_{1},f_{2},\ldots ,f_{k}\right\}=\mathrm{Topk}\left(f_{*}\in \left\{1,\ldots ,\left.\left[\frac{T}{2}\right]\right\}\right.(A)\right)$$

where Topk denotes the operation of retrieving the indices of the top *k* maximum values.

Period Length Calculation: Based on the inverse relationship between frequency and period, the period length corresponding to each selected dominant frequency is calculated as follows:

(3) $$p_{i}=\left[\frac{T}{f_{i}}\right],i=1,2,\ldots ,k$$

The above formulation is summarized as follows:

(4) $$A,\left\{f_{1},\ldots ,f_{k}\right\},\left\{p_{1},\ldots p_{k}\right\}=\mathrm{Period}(X_{1D})$$

Since the period length *p_i_* may not be able to evenly divide the original sequence length *T*, direct reshaping would result in an incomplete final period. To address this issue, TimesNet performs zero-padding along the temporal dimension to extend the sequence length:

(5) $$T^{\prime }=p_{i}\times \left\lceil \frac{T}{p_{i}}\right\rceil$$

After zero-padding, the extended sequence length becomes divisible by the selected period length, ensuring that each period has the same length. The padded one-dimensional time series is then reconstructed into a regular two-dimensional periodic representation through the reshaping operation. The reshaping formula is given as follows:

(6) $$X_{2D}^{i}={\mathrm{Reshape}}_{p_{i},\frac{T^{\prime }}{p_{i}}}(\mathrm{Padding}(X_{1D})),i\in \left\{1,\ldots ,k\right\}$$

In the resulting two-dimensional representation, the row direction corresponds to different periods, reflecting inter-period variations, while the column direction corresponds to time steps within a single period, reflecting intra-period variations. This structure naturally contains two types of local dependencies: adjacent elements along the column describe short-term fluctuations within a period, whereas adjacent elements along the row represent trend evolution across periods. Therefore, 2D convolutional kernels can be employed to simultaneously model intra-period fluctuations and inter-period trends, thereby enhancing the representation capability of the model for complex temporal structures. Ultimately, a group of two-dimensional representations under different period scales is generated for subsequent feature extraction [24,25,26].

#### 3.2.2. Modular Design

**Figure 2:**
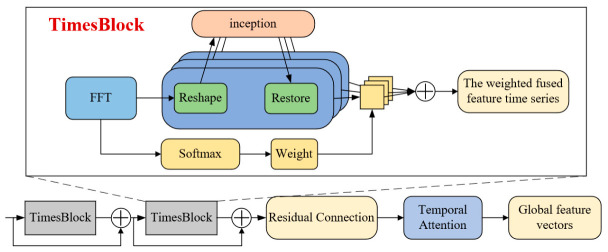
Architecture diagram of the TimesNet model. The colors are used only to visually distinguish different functional modules and do not indicate a strict one-to-one correspondence or additional technical meaning. The dashed lines indicate an enlarged view of the internal structure of the TimesBlock.

As shown in Figure 2, the TimesNet model constructs a deep network by stacking multiple TimesBlock modules, which progressively extract and enhance time-series features. Taking one TimesBlock as an example, the output sequence from the previous module is used as its input. The module first identifies dominant periods, transforms the one-dimensional sequence into two-dimensional representations and then extracts temporal features through two-dimensional convolution:

(7) $$X_{1D}^{l}=\mathrm{TimesBlock}(X_{1D}^{l-1})+X_{1D}^{l-1}$$

**Figure 3:**
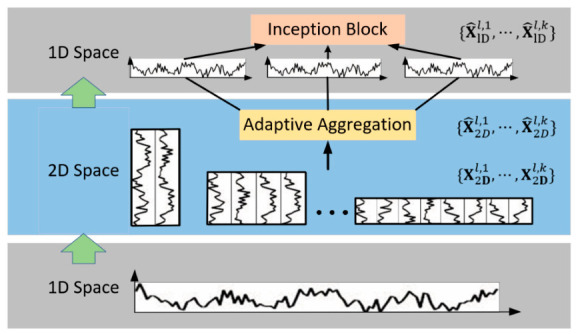
TimesBlock module. The gray regions represent the 1D feature spaces, while the blue region represents the 2D feature space. The orange block denotes the Inception Block, and the yellow block denotes the adaptive aggregation operation. The black arrows indicate the direction of feature propagation and processing, whereas the green arrows indicate the transformations between the 1D and 2D spaces, including the reshaping from 1D to 2D and the restoration from 2D to 1D. The ellipsis between the 2D feature maps indicates that the intermediate period-specific feature representations are omitted for clarity.

The output of each TimesBlock is passed to the next module, enabling the features to be progressively refined and enhanced through the stacked network. As illustrated in Figure 3, each TimesBlock mainly consists of the following sub-processes:

(1) 1D-to-2D Transformation

First, the module identifies the dominant periodic components from the input one-dimensional time series. Based on the detected periods, the input sequence is padded when necessary and then reshaped into multiple two-dimensional representations. This process is formulated as follows:

(8) $$A^{l-1},\left\{f_{1},\ldots ,f_{k}\right\},\left\{p_{1},\ldots p_{k}\right\}=\mathrm{Period}(X_{1D}^{l-1})$$

(9) $$X_{2D}^{l,i}={\mathrm{Reshape}}_{p_{i},\frac{T^{\prime }}{p_{i}}}(\mathrm{Padding}(X_{1D}^{l-1})),i\in \left\{1,\ldots ,k\right\}$$

(2) 2D Feature Extraction

For each generated two-dimensional representation, a two-dimensional convolutional operation is performed to capture local dependencies within and across periods. The feature extraction process is expressed as follows:

(10) $${\overset{⌢}{X}}_{2D}^{l,i}=\mathrm{Inception}(X_{2D}^{l,i})$$

(3) 2D-to-1D Transformation

After convolutional processing, the two-dimensional representation is transformed back into a one-dimensional form for subsequent cross-period fusion:

(11) $${\overset{⌢}{X}}_{1D}^{l,i}=\mathrm{Trunc}({\mathrm{Reshape}}_{1,(p_{i},\frac{T^{\prime }}{p_{i}})}({\overset{⌢}{X}}_{2D}^{l,i})),i\in \left\{1,\ldots ,k\right\}$$

where the truncation operation removes the padded zeros and restores the valid temporal length.

(4) Adaptive Fusion

After obtaining the one-dimensional outputs under different period scales, the model fuses them into a unified temporal representation. The fusion weights are determined according to the frequency-domain amplitudes obtained during period detection. Period components with higher amplitudes are assigned larger weights, indicating their greater contribution to the final representation. The adaptive fusion process is given as follows:

(12) $${\overset{⌢}{A}}_{f_{1}}^{l-1},\ldots ,{\overset{⌢}{A}}_{f_{k}}^{l-1}=\mathrm{Softmax}(A_{f_{1}}^{l-1},\ldots ,A_{f_{k}}^{l-1})$$

(13) $$X_{1D}^{l}=\sum_{i=1}^{k}{\overset{⌢}{A}}_{f_{i}}^{l-1}\times {\overset{⌢}{X}}_{1D}^{l,i}$$

After global periodicity modeling by the TimesNet branch, a temporal feature sequence is obtained. Since the sequence still retains the temporal dimension, it cannot be directly used for final classification. Therefore, a temporal attention mechanism is introduced to assign adaptive weights to different time steps and aggregate them into a fixed-dimensional global representation:

(14) $$f_{\mathrm{TimesNet}}=\sum_{t=1}^{T}a_{t}h_{t}^{\left(L\right)}$$

where the feature at each time step is assigned an adaptive attention weight generated by learnable parameters, enabling the model to focus on key time periods with strong discriminative power. The weighted aggregation produces a context vector, which is then mapped by a fully connected layer into a unified semantic space to obtain the final global periodic feature of the TimesNet branch.

### 3.3. CNN Model

This paper employs a 1D Convolutional Neural Network (1D-CNN) model to extract local temporal dynamics and high-frequency transient features from raw multivariate time series. Starting from the raw input signals before embedding, this branch stacks several operations, including small convolutional kernels, batch normalization, nonlinear activation and pooling. While compressing the temporal length, it progressively abstracts local patterns layer by layer, effectively capturing short-term anomalous behaviors such as current surges and voltage spikes. Therefore, the CNN branch serves as an important complement to the global periodic structures modeled by the TimesNet branch. The input of this branch is organized as a three-dimensional batch-time-channel representation, where *B*, *T* and *C* denote the batch size, the number of time steps and the number of variable channels, respectively.

First, the input representation is transposed along the channel and temporal dimensions to adapt to the standard input format of 1D convolution:

(15) $$X^{\left(0\right)}=\mathrm{permute}(X_{1D},(0,2,1))$$

where permute denotes the dimension rearrangement operation.

Subsequently, hierarchical feature extraction is performed through three sequential convolutional modules:

The first two convolutional blocks share an identical structure, each consisting of a 1D convolutional layer (Conv1D*_k_*_=3_), batch normalization (BN) and the RELU activation function. To achieve temporal dimension compression and expand the receptive field, each convolutional block is immediately followed by a max-pooling layer (MaxPool) with a stride of 2. The feature extraction process at the *i*-th level (*i* = 1,2) can be expressed as

(16) $$X^{\left(i\right)}={\mathrm{MaxPool}}_{S=2}\left(\mathrm{ReLU}\left({\mathrm{BN}}_{i}\left(\mathrm{Conv}1D_{k=3}\left(X^{\left(i-1\right)}\right)\right)\right)\right)$$

The third-level convolutional block focuses on extracting high-level semantic features. It includes convolution, batch normalization and activation operations but omits pooling to preserve finer local structures:

(17) $$X^{\left(3\right)}=\mathrm{ReLU}\left({\mathrm{BN}}_{3}\left(\mathrm{Conv}1D_{k=3}\left(X^{\left(2\right)}\right)\right)\right)$$

1D dropout is then applied to suppress overfitting, where *p*_1_ is the preset dropout rate:

(18) $$X^{\left(4\right)}={\mathrm{Dropout}}_{p_{1}}\left(X^{\left(3\right)}\right)$$

The output feature map is flattened into a one-dimensional feature vector:

(19) $$f_{\mathrm{CNN}}=\mathrm{Flatten}\left(X^{\left(4\right)}\right)\in {\mathbb{R}}^{B\times d_{f}}$$

where Flatten denotes the operation used to convert a multi-dimensional feature map into a one-dimensional feature vector, and *d_f_* represents the flattened feature dimension.

**Figure 4:**
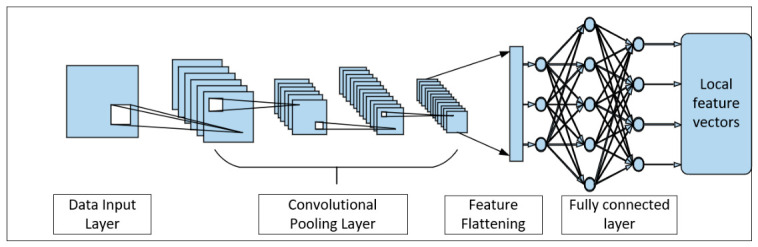
Architecture diagram of the CNN model. The blue rectangle on the left represents the input data. The stacked rectangles represent multichannel feature maps generated by the convolution and pooling operations, with their decreasing sizes indicating progressive feature downsampling. The narrow vertical rectangle represents the flattened feature vector. The circles and connecting lines represent the neurons and their connections in the fully connected layers, respectively, while the rounded rectangle on the right represents the extracted local feature vectors.

Finally, a two-layer fully connected network is employed for nonlinear projection:

(20) $$h^{\left(1\right)}=\mathrm{ReLU}\left(w_{1}f_{\mathrm{CNN}}+b_{1}\right)$$

(21) $$h^{\left(2\right)}={\mathrm{Dropout}}_{p_{2}}\left(h^{\left(1\right)}\right)$$

(22) $$Z_{CNN}=w_{2}h^{\left(2\right)}+b_{2}$$

where ***w***_1_, ***w***_2_ are learnable weight matrices, *p*_2_ is the preset dropout rate, ***b***_1_, ***b***_2_ are bias terms and the final output ***Z***_CNN_ serves as the local temporal feature representation, to be fused with the global periodic features later. Figure 4 illustrates the schematic diagram of the CNN model adopted in this paper [27].

### 3.4. Feature Fusion and Classification Decision

The global periodic feature ***Z***_TimesNet_ output by the TimesNet branch and the dimension-aligned local feature ***Z***_CNN_ output by the CNN branch are concatenated along the feature dimension to form a joint representation ***Z***_Splice_:

(23) $$Z_{\mathrm{splice}}=[Z_{\mathrm{TimesNet}};Z_{\mathrm{CNN}}]$$

To enhance the nonlinear representation capability of the fused features and prevent overfitting, a small Multi-Layer Perceptron (MLP) is introduced as the fusion network:

(24) $$Z_{\mathrm{fused}}=\mathrm{MLP}\left(Z_{\mathrm{Splice}}\right)$$

This MLP incorporates RELU activation and dropout regularization, ultimately outputting the discriminative fused feature.

Finally, a lightweight classification head maps the final feature vector ***Z***_fused_ to the category logits space and the Softmax function is applied to obtain the diagnostic probability distribution for each category:

(25) $$\overset{⌢}{p}=\mathrm{Softmax}\left(W_{C}Z_{\mathrm{fused}}+b_{c}\right)$$

where ***W_c_*** is the weight matrix of the classification head and ***b_c_*** is the bias vector of the classification head.

Ultimately, the category with the highest predicted probability is selected as the diagnostic result:

(26) $$\overset{⌢}{c}=\arg\;\underset{y}{\max}\;\overset{⌢}{p}$$

where the argmax operation selects the class index with the largest predicted probability among all diagnostic categories.

### 3.5. Summary of the Proposed Method

This subsection summarizes the proposed deep temporal classification method based on the dual-branch TimesNet-CNN architecture for multi-fault diagnosis of boost choppers. First, an embedding layer generates the initial feature representation, which is then fed in parallel into two complementary branches: the TimesNet branch utilizes the Fast Fourier Transform (FFT) to adaptively identify the dominant periods within the signal and models long-range periodic dependencies on a 2D periodic view; meanwhile, the CNN branch extracts local transient and short-term anomalous features by stacking 1D convolutional layers. The sequential features output by TimesNet are aggregated into a global vector via a temporal attention mechanism and then concatenated with the local features from the CNN under a unified dimension. Finally, a lightweight classification head maps the fused features into diagnostic probabilities for various system states.

Compared with a single-branch architecture, this design improves the representation of HS fault signals by jointly modeling global periodic evolution and local transient disturbances, thereby enhancing the model’s ability to distinguish multi-scale fault patterns.

## 4. Experiments

### 4.1. Data Acquisition and Sample Generation

The raw operational data used in this study originate from real-time communication packets of the high-speed maglev train’s actual operating platform, rather than from numerical simulation or manually generated data. Using standard SQL query interfaces, the six core electrical parameters mentioned above are read in batches from TDengine in chronological order for subsequent offline fault diagnosis experiments, including model training, validation and testing. The data acquisition cycle is set to 500 ms, corresponding to a sampling frequency of 2 Hz. All parameters have undergone pre-processing operations, including unit normalization, outlier filtering and clock synchronization, prior to being written into the database, ensuring that the data quality meets the modeling requirements.

**Figure 5:**
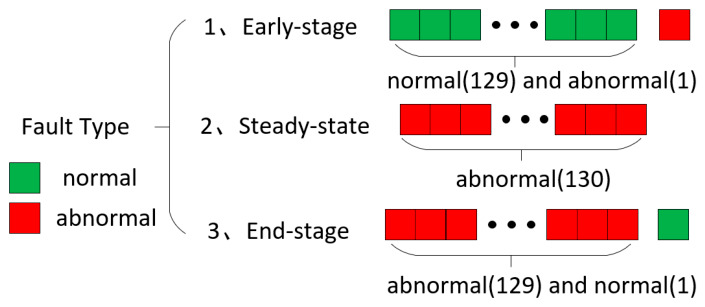
Transition-boundary-enhanced sliding-window strategy. The ellipsis indicates that the intermediate time steps are omitted for clarity. Only representative elements are shown, while the actual input sequence contains 130 time steps.

To adapt the raw sampling data to deep learning-based multivariate time-series fault diagnosis tasks, this paper employs a sliding-window mechanism for sample reconstruction. The constructed samples are directly generated from the real-vehicle operational records and are not obtained by modifying the outputs of any baseline models. When constructing fault samples, the dataset includes not only steady-state fault samples that are entirely within the stable fault phase but also transition-state samples from the initial fault onset and final recovery stages. This strategy is used to enhance the model’s ability to identify fault boundaries and dynamic evolution processes. The transition-boundary-enhanced sliding-window strategy is illustrated in Figure 5.

Considering the dynamic response characteristics and fault evolution patterns of the boost chopper system, the time-window length is set to 130 consecutive time steps. Since the sampling interval is 0.5 s, each sample covers 65 s of historical operational data. Each sample is organized as a two-dimensional time-channel representation with dimensions of [130, 6], where 130 denotes the number of time steps and 6 represents the selected electrical parameter channels. In the sliding-window construction process, the sliding step is set to 1 time step, corresponding to 0.5 s. Therefore, two adjacent windows overlap by 129 time steps, and the overlap ratio is approximately 99.23%. The high-overlap sliding-window strategy is adopted to preserve the continuity of fault evolution and to increase the number of available samples for rare system states.

Due to significant differences in the actual occurrence frequencies of different system states, the original constructed samples suffer from a class-imbalance problem, which may affect the recognition performance for rare fault categories. To address this issue and reduce the risk of data leakage caused by highly overlapping windows, the training-candidate samples and test samples are constructed as separate feature sample files and used for different stages of the experiment. The class-balanced training–validation subset is constructed only from the training-candidate samples, while the test samples retain the original sample distribution of the 11 system states and are used only for final model evaluation.

To further improve the reproducibility and credibility of the experimental evaluation, the statistics of the constructed dataset and data partition are summarized in Table 2. For the 11 system states considered in this study, the training-candidate sample set and the test sample set each contain 1,757,025 window samples. For model training, 1000 samples are randomly selected from each system state to construct the class-balanced training–validation subset. The selected samples are then divided into training and validation sets at a ratio of 9:1 using stratified sampling. Therefore, each system state contains 900 training samples and 100 validation samples. Although the training-candidate samples and test samples have the same class distribution, they are separately constructed feature sample files and are used for different stages of the experiment. The class-balanced training–validation subset is used for model training and model selection, whereas the test samples are used only for final performance evaluation.

### 4.2. Model Training

**Figure 6:**
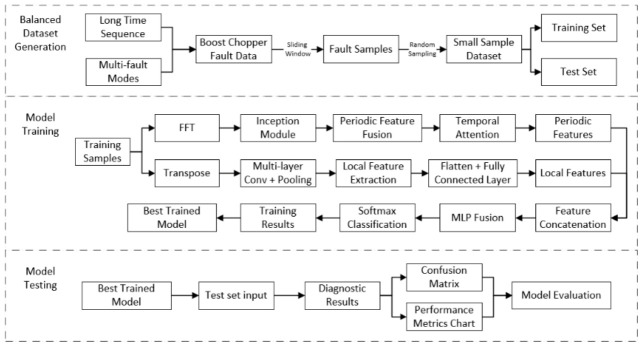
Fault diagnosis flowchart.

This experiment employs a dual-branch fused deep learning model for the fault diagnosis and classification task of the boost chopper system. The fault diagnosis flowchart is shown in Figure 6. The overall process consists of three main stages: balanced dataset generation, model training and model testing. In the model training stage, the training samples are fed into two parallel branches. The TimesNet branch extracts global periodic features through FFT-based period detection, Inception-based feature extraction, periodic feature fusion and temporal attention aggregation. Meanwhile, the CNN branch extracts local transient features through transposition, multi-layer convolution, pooling and fully connected mapping. The two types of features are concatenated and fused by the MLP module, and the Softmax classifier outputs the training classification results. The model with the highest validation accuracy is saved as the best trained model for subsequent testing.

In the TimesNet branch, the input multivariate time series with a length of 130 and 6 channels is first encoded through a DataEmbedding layer into position-independent embeddings, with the embedding dimension set to 64. It is then processed by a stack of two TimesBlock modules. Each TimesBlock contains an FFT-based period detection operation to automatically detect the top two significant periodic components and reshapes the original one-dimensional sequence into a two-dimensional periodic representation according to the detected period length. In this way, the model can explicitly represent multi-scale periodic patterns within the time series. Subsequently, the Inception_Block_V1 convolution module is adopted for multi-scale feature extraction, enhancing the model’s ability to perceive non-stationary dynamic behaviors. To replace traditional Global Average Pooling (GAP), a temporal attention mechanism is introduced to adaptively weight the features of different time steps, thereby enabling the model to focus on periods with stronger discriminative power.

In the CNN branch, the raw input is first transposed along the channel and temporal dimensions and then sequentially passes through three 1D convolutional layers equipped with batch normalization (BN) and RELU activation functions. Max-pooling and dropout regularization are further introduced to compress the temporal dimension and improve the robustness of feature extraction. Finally, the output feature map of the CNN branch is flattened into a high-dimensional feature vector.

The features extracted by the TimesNet branch and CNN branch are independently mapped to a 512-dimensional feature space through fully connected layers. Then, the two types of features are concatenated and further processed by a lightweight MLP fusion module to enhance nonlinear feature interaction. The fused feature is finally fed into an 11-dimensional classification head to output the diagnostic result.

During the model training process, the Adam optimizer is employed with an initial learning rate of 1 × 10^−3^, and L2 regularization with a weight decay of 1 × 10^−4^ is introduced to reduce overfitting. Meanwhile, the learning rate is halved if the validation accuracy does not improve for 10 consecutive epochs. The total number of training epochs is set to 100, with a batch size of 64. After each training epoch, the model performance is evaluated on the validation set, and the model with the highest validation accuracy is saved as the best trained model for subsequent testing.

### 4.3. Model Testing and Evaluation

After model training, the model with the highest validation accuracy is selected as the best trained model for subsequent testing. During the testing stage, the separately constructed test samples are fed into the trained TimesNet-CNN model without any parameter update. The test samples are not involved in the class-balanced training–validation subset construction or random training–validation split or model selection and are used only for final performance evaluation. Each test sample first passes through the TimesNet branch and CNN branch in parallel to extract global periodic features and local transient features, respectively. The two types of features are then concatenated and fused by the MLP module, and the Softmax classifier outputs the diagnostic probability distribution over 11 system states. The category with the highest probability is selected as the final diagnostic result.

To evaluate the diagnostic performance, the predicted labels are compared with the true labels of the test samples. The confusion matrix is used to visualize the classification results and misclassification distribution of each fault category. In addition, precision, recall and F1-score are adopted as quantitative evaluation metrics to assess the diagnostic accuracy, fault coverage ability and overall balanced performance of different models.

### 4.4. Model Comparison

To systematically verify the effectiveness and superiority of the proposed TimesNet-CNN dual-branch model in the fault diagnosis task of boost choppers, this paper designs ablation experiments and comparative experiments under consistent data partition and training settings. For fairness, all baseline models and ablated variants are independently implemented, trained and tested under the same dataset partition and evaluation criteria, rather than being generated by modifying the prediction results of other models.

The ablation experiments are conducted to evaluate the contribution of each branch in the proposed dual-branch architecture. Specifically, two ablated variants are constructed: a TimesNet-only model, in which the CNN branch is removed, and a CNN-only model, in which the TimesNet branch is removed. By comparing these two variants with the complete TimesNet-CNN model, the effectiveness of the complementary dual-branch design can be verified.

The comparative experiments are designed to further evaluate the performance of the proposed method against representative baseline models. Informer [28] and ResNet [29] are selected as comparison models. Informer represents a Transformer-based long-sequence modeling method, while ResNet represents a typical deep residual feature extraction network. By comparing the proposed model with these representative methods, the advantages of the TimesNet-CNN dual-branch architecture in multi-scale temporal feature representation can be further demonstrated.

All models are trained and tested under the same data partition and evaluation criteria. The diagnostic performance is analyzed using training and validation accuracy curves, confusion matrices, precision, recall and F1-score.

## 5. Results

### 5.1. Experimental Results of the Proposed Dual-Branch Model

**Figure 7:**
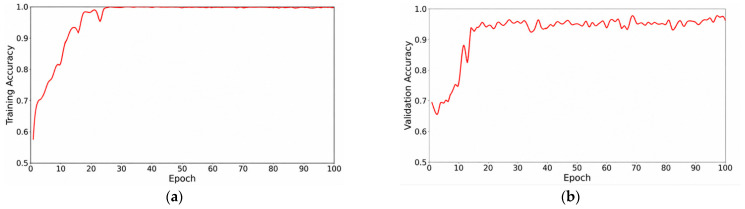
Training and validation accuracy of the proposed TimesNet-CNN dual-branch model. (**a**) Training accuracy; (**b**) validation accuracy.

To first demonstrate the diagnostic performance of the proposed TimesNet-CNN dual-branch model itself, this subsection presents the experimental results of the complete model before conducting ablation and comparative analyses. The training and validation accuracy curves of the proposed model are shown in Figure 7. As can be seen from Figure 7, the proposed dual-branch model exhibits rapid convergence during the training process, and the training accuracy gradually approaches a stable high level as the number of epochs increases. Meanwhile, the validation accuracy also increases rapidly in the early training stage and remains at a relatively high level after convergence, indicating that the model has good learning stability and does not show obvious overfitting.

**Figure 8:**
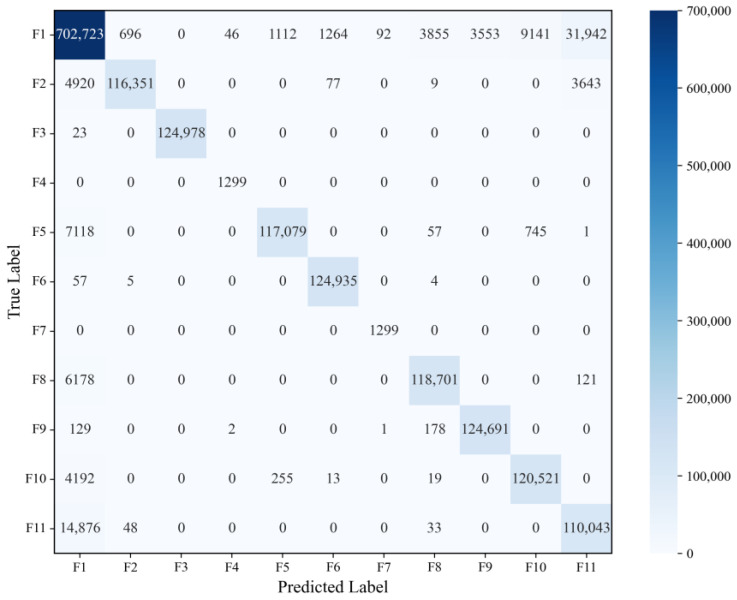
Confusion matrix of the proposed TimesNet-CNN dual-branch model.

**Figure 9:**
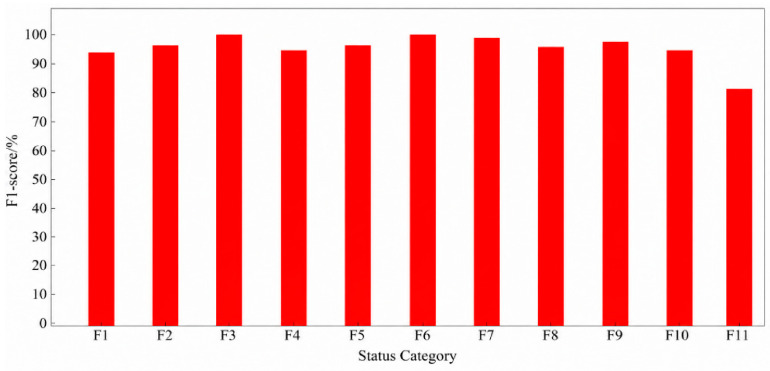
F1-score of the proposed TimesNet-CNN dual-branch model.

To further evaluate the diagnostic performance of the proposed model on the independent test set, the confusion matrix and F1-score results are shown in Figure 8 and Figure 9, respectively. The confusion matrix in Figure 8 shows that the predicted results are mainly concentrated along the diagonal, indicating that most samples can be correctly classified into their corresponding system states. The F1-score results in Figure 9 further demonstrate that the proposed model achieves high diagnostic performance across most categories. Since F1-score comprehensively reflects the balance between precision and recall, these results indicate that the proposed method maintains both strong diagnostic accuracy and fault coverage ability. These results verify the effectiveness of the proposed TimesNet-CNN dual-branch architecture in extracting and fusing global periodic features and local transient features from multivariate time-series data.

To further evaluate the deployment feasibility of the proposed TimesNet-CNN dual-branch model, its computational efficiency is analyzed in terms of parameter number, model size, GPU memory consumption and inference time. The inference time is calculated as the average forward-propagation time of a single test sample without parameter updating. The results are summarized in Table 3.

As shown in Table 3, the proposed TimesNet-CNN model contains 23.01 million parameters, including 23.01 million trainable parameters. The model size is 89.03 MB, and the peak GPU memory consumption during inference is 261.50 MB. The average inference time for a single test sample is 13.10 ms. Although the proposed framework integrates the FFT-based period extraction operation, TimesBlocks, temporal attention mechanism, CNN feature extraction branch and feature fusion module, its inference cost remains moderate. Since the input sample length is fixed and the diagnostic process only involves forward propagation after offline training, the trained model can perform fast diagnosis on newly collected monitoring data. These results indicate that the proposed TimesNet-CNN framework has potential deployment feasibility for intelligent fault diagnosis and condition monitoring of high-speed maglev train onboard power systems.

### 5.2. Ablation Experiments

**Figure 10:**
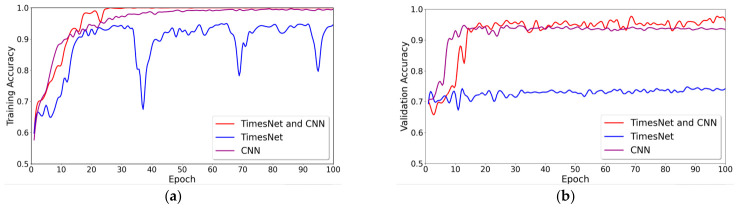
Accuracy comparison in the ablation experiments. (**a**) Training accuracy comparison; (**b**) validation accuracy comparison.

Figure 10 presents the accuracy comparison during the training and validation processes of the ablation experiments. From the comparative analysis of training and validation, it can be observed that the TimesNet-only model exhibits relatively lower accuracy. The CNN-only model shows rapid growth in the early stages of training and validation but tends to plateau in the later stages. In contrast, the TimesNet-CNN dual-branch model reaches its peak performance earliest during the training process and its validation accuracy consistently improves with training epochs, ultimately reaching approximately 96.5%, which outperforms the single-branch models.

**Figure 11:**
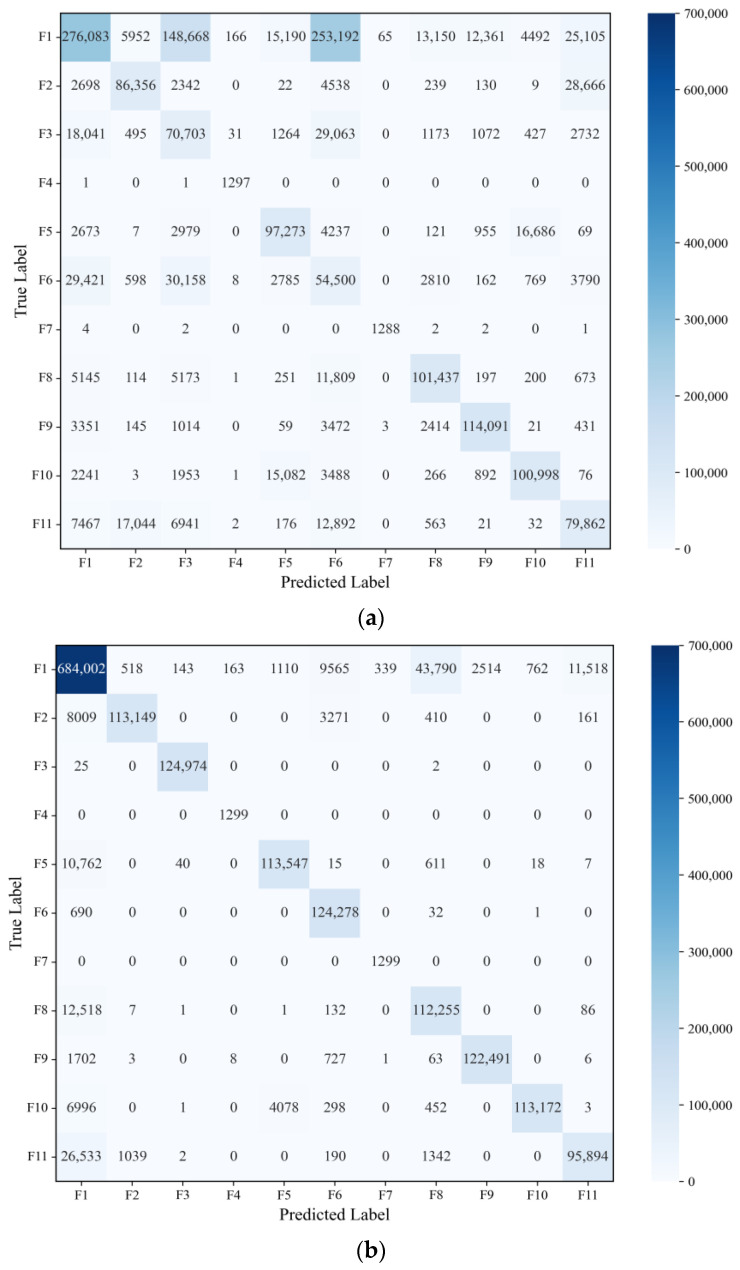
Confusion matrices of ablated models. (**a**) TimesNet-only model; (**b**) CNN-only model.

During the testing phase, the single-branch ablated models are further evaluated using the independent test set. Since the confusion matrix of the complete TimesNet-CNN dual-branch model has already been presented in Figure 8, only the confusion matrices of the two ablated variants are shown in Figure 11 to avoid repeated presentation. As shown in Figure 11, the TimesNet-only model exhibits relatively severe misclassification for certain system states, such as F1, F3 and F6, indicating that the global periodic branch alone cannot sufficiently capture short-term abnormal disturbances. The CNN-only model performs better than the TimesNet-only model in several categories, but it still shows cross-confusion in the identification of categories such as F1 and F8. Compared with the two single-branch variants, the complete dual-branch model shown in Figure 8 presents a more diagonally dominant confusion matrix, with higher diagnostic accuracy for each category and fewer misclassified samples, demonstrating stronger discriminative capability.

**Figure 12:**
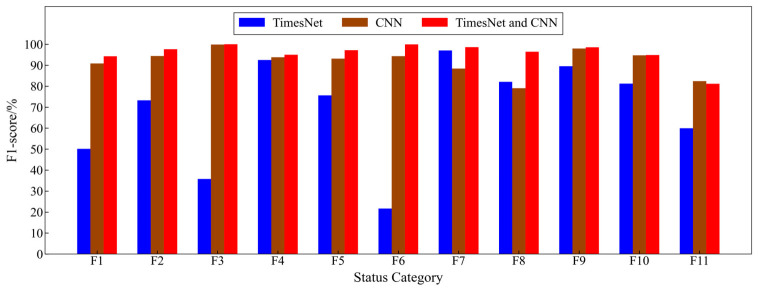
F1-score comparison of models in the ablation experiments.

As shown in Figure 12, the proposed TimesNet-CNN dual-branch model achieves higher F1-score values than the TimesNet-only and CNN-only variants in most system states. The TimesNet-only model shows relatively weak performance in some categories, indicating that global periodic modeling alone is insufficient to fully capture local transient fault features. Although the CNN-only model improves the recognition performance of some categories, its overall F1-score is still lower than that of the complete dual-branch model. This further confirms that the fusion of global periodic features and local transient features can improve the balanced diagnostic performance of the model.

In summary, the ablation experiments results demonstrate that the proposed TimesNet-CNN dual-branch structure significantly outperforms the TimesNet-only and CNN-only variants in the fault diagnosis and classification task. It exhibits faster convergence speed and stronger stability during the training and validation phases, while simultaneously demonstrating superior generalization capability during the testing phase.

### 5.3. Comparative Experiments

**Figure 13:**
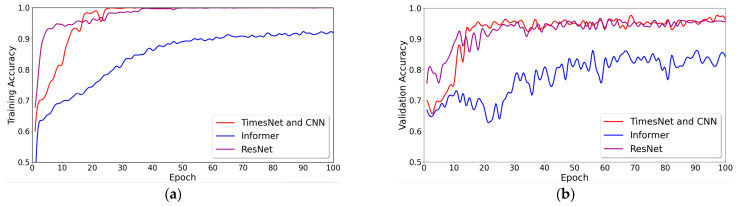
Accuracy comparison in the comparative experiments. (**a**) Training accuracy comparison; (**b**) validation accuracy comparison.

As shown in Figure 13, there are significant differences in the performance of each model during the training and validation processes. The Informer model exhibits slow convergence and relatively low final accuracy. The ResNet model converges quickly in the early stages of training and validation, but its subsequent growth is sluggish, requiring more iterations. In contrast, the dual-branch model not only reaches its performance peak earliest but also achieves higher accuracy during the validation phase, indicating that its generalization capability outperforms that of the Informer and ResNet models.

**Figure 14:**
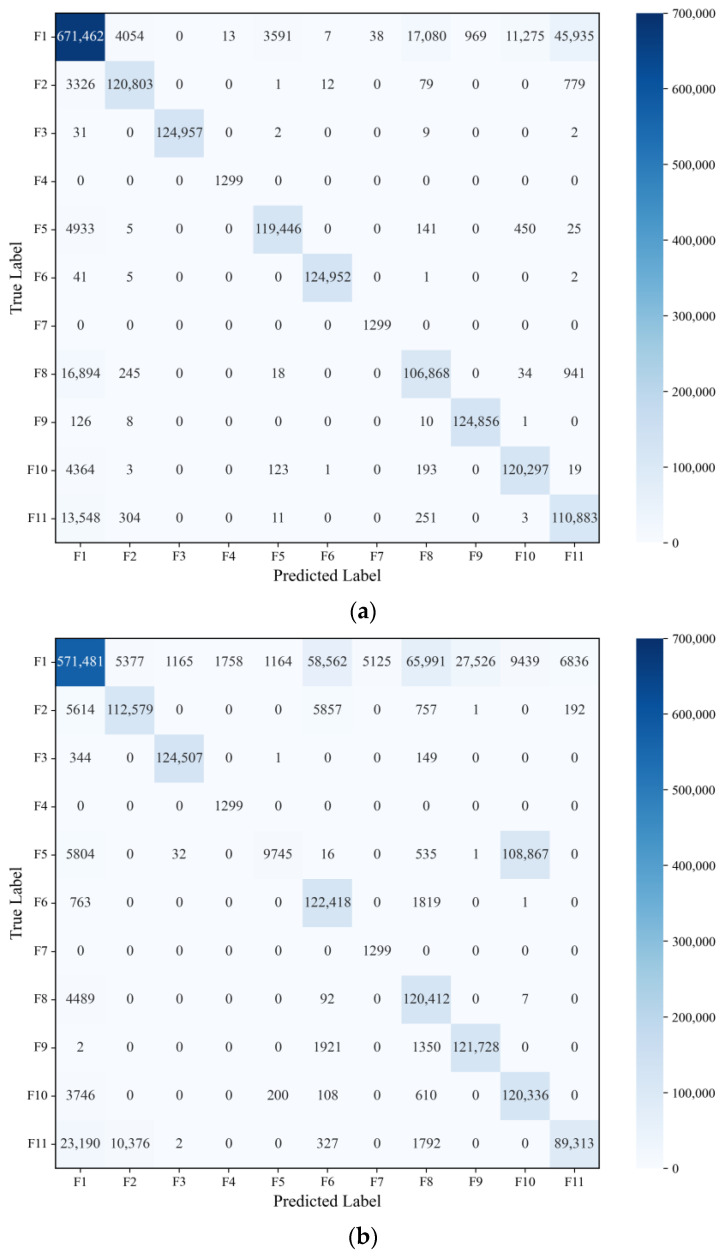
Confusion matrices of baseline models in the comparative experiments. (**a**) ResNet model; (**b**) Informer model.

During the testing phase, confusion matrices are generated for the ResNet and Informer models based on the independent test set, as shown in Figure 14. Since the confusion matrix of the proposed TimesNet-CNN model has already been shown in Figure 8, it is not repeated here. As shown in Figure 14, the ResNet model frequently misjudges the normal state F1 and often misclassifies it as F8, F10 or F11. The Informer model also shows obvious misclassification of F1 into multiple fault categories, indicating relatively weak boundary recognition capability. Compared with these baseline models, the proposed dual-branch model achieves more accurate and stable classification results, as demonstrated in Figure 8.

**Figure 15:**
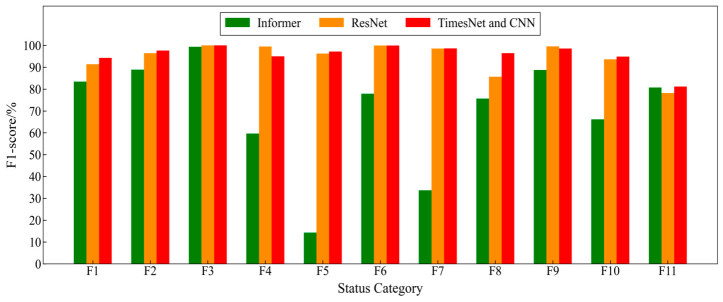
F1-score comparison of models in the comparative experiments.

As shown in Figure 15, the proposed TimesNet-CNN dual-branch model achieves higher F1-score values than the Informer and ResNet models in most system states. The Informer model shows relatively limited performance in this task, which indicates that its long-sequence modeling structure may not be sufficiently effective for distinguishing the multi-scale fault patterns of the boost chopper. The ResNet model performs better than Informer in several categories, but its F1-score is still generally lower than that of the proposed model. These results demonstrate that the proposed dual-branch architecture has stronger capability in multi-scale temporal feature representation and fault pattern discrimination.

Table 4 presents the overall performance of the five models across the 11 system states, including precision, recall and F1-score.

The experimental results demonstrate that the proposed dual-branch model outperforms the TimesNet, CNN, Informer and ResNet models in the fault diagnosis task, fully validating the effectiveness and superiority of this dual-branch architecture for the current fault diagnosis task.

## 6. Conclusions

(1) A fault diagnosis method for the boost chopper of high-speed maglev trains is proposed based on a TimesNet-CNN dual-branch architecture. By parallelly fusing the global periodic modeling capability of the TimesNet branch with the local transient feature extraction advantage of the CNN branch, this method effectively resolves the challenge wherein a single network architecture struggles to synchronously capture multi-scale temporal features in scenarios characterized by coexisting fast-varying transients and long-period fluctuations of high-speed maglev trains.

(2) A feature-adaptive aggregation strategy based on temporal attention and a lightweight MLP fusion decision module are designed. This mechanism can dynamically focus on key periods with strong discriminative power, performing nonlinear mapping and deep fusion of global periodic feature vectors and local transient feature vectors, which significantly enhances the diagnostic system’s sensitivity to fault evolution patterns and boundary states.

(3) A balanced dataset comprising 11 categories (10 fault types + 1 normal state) is constructed based on real-vehicle operation data. Ablation experiments and comparative experiments verify that the proposed model outperforms single-branch models, as well as Informer and ResNet models, in terms of precision, recall and F1-score, demonstrating excellent multi-scale fault feature decoupling capability.

(4) The proposed method still has certain limitations. It is mainly applicable to supervised fault diagnosis scenarios with known fault categories and sufficient labeled multivariate electrical data. Its generalization ability under different train types, operating conditions and unknown or compound faults still needs to be further verified. In future work, the proposed framework can be extended by incorporating domain adaptation, transfer learning and incremental learning strategies to improve its adaptability to different operating environments and newly emerging fault patterns. In addition, by combining the proposed diagnostic model with online monitoring data and historical maintenance records, it can be further developed into an intelligent fault diagnosis and predictive maintenance system for key electrical components of high-speed maglev trains.

## Figures and Tables

**Table 1:** Types of system states.

Label	System State Type
F1	Normal Operating State
F2	Abnormal HS Equipment Status
F3	HS Output Current Average Exceeds Limit
F4	Battery Current Exceeds Limit
F5	Battery Voltage Exceeds Limit
F6	Abnormal 440 V Battery Status
F7	More Than 2 LIG Phases Idling
F8	LIG Output Current Exceeds Limit (With External Power)
F9	LIG Output Current Exceeds Limit (Without External Power)
F10	Non-zero LIG Current When Vehicle is Lowered
F11	Abnormal External Power Supply Voltage

**Table 2:** Statistics of the constructed dataset and data partition.

Label	System State	Training-Candidate Samples Before Balancing	Balanced Training–Validation Samples	Training Samples	Validation Samples	Test Samples
F1	Normal Operating State	754,424	1000	900	100	754,424
F2	Abnormal HS Equipment Status	125,000	1000	900	100	125,000
F3	HS Output Current Average Exceeds Limit	125,001	1000	900	100	125,001
F4	Battery Current Exceeds Limit	1299	1000	900	100	1299
F5	Battery Voltage Exceeds Limit	125,000	1000	900	100	125,000
F6	Abnormal 440 V Battery Status	125,001	1000	900	100	125,001
F7	More Than 2 LIG Phases Idling	1299	1000	900	100	1299
F8	LIG Output Current Exceeds Limit (With External Power)	125,000	1000	900	100	125,000
F9	LIG Output Current Exceeds Limit (Without External Power)	125,001	1000	900	100	125,001
F10	Non-zero LIG Current When Vehicle is Lowered	125,000	1000	900	100	125,000
F11	Abnormal External Power Supply Voltage	125,000	1000	900	100	125,000
Total	—	1,757,025	11,000	9900	1100	1,757,025

**Table 3:** Computational efficiency of the proposed TimesNet-CNN dual-branch model.

Model	Total Params	Trainable Params	Model Size	GPU Memory	Inference Time
Proposed TimesNet-CNN	23.01 M	23.01 M	89.03 MB	261.50 MB	13.10 ms

**Table 4:** Results comparison of model indicators.

Model	Precision/%	Recall/%	F_1_-Score/%
Proposed Model	95.3	95.0	95.1
TimesNet	81.9	55.9	66.4
CNN	91.9	91.4	91.5
Informer	84.4	79.4	78.0
ResNet	93.0	92.6	92.7

## Data Availability

The data are not publicly available due to privacy or ethical restrictions (commercial confidentiality).
